# Effects of cues to event segmentation on subsequent memory

**DOI:** 10.1186/s41235-016-0043-2

**Published:** 2017-01-30

**Authors:** David A. Gold, Jeffrey M. Zacks, Shaney Flores

**Affiliations:** 1 Krembil Neuroscience Centre, University Health Network, Toronto Western Hospital, Neuropsychology Clinic, 4F-409, 399 Bathurst St., Toronto, ON M5T 2S8 Canada; 2 Department of Psychological and Brain Sciences, Washington University in St. Louis, St Louis, MO 63130 USA

**Keywords:** Event cognition, Cognitive aging, Event segmentation theory, Event horizon model

## Abstract

To remember everyday activity it is important to encode it effectively, and one important component of everyday activity is that it consists of events. People who segment activity into events more adaptively have better subsequent memory for that activity, and event boundaries are remembered better than event middles. The current study asked whether intervening to improve segmentation by cuing effective event boundaries would enhance subsequent memory for events. We selected a set of movies that had previously been segmented by a large sample of observers and edited them to provide visual and auditory cues to encourage segmentation. For each movie, cues were placed either at event boundaries or event middles, or the movie was left unedited. To further support the encoding of our everyday event movies, we also included post-viewing summaries of the movies. We hypothesized that cuing at event boundaries would improve memory, and that this might reduce age differences in memory. For both younger and older adults, we found that cuing event boundaries improved memory—particularly for the boundaries that were cued. Cuing event middles also improved memory, though to a lesser degree; this suggests that imposing a segmental structure on activity may facilitate memory encoding, even when segmentation is not optimal. These results provide evidence that structural cuing can improve memory for everyday events in younger and older adults.

## Significance

Many of us would like to be able to remember events better, and memory problems are a particular concern of older adults. For structured materials such as shopping lists and colleagues’ names one can use techniques such as the method of loci or spaced retrieval practice. However, often what we want to remember is not structured lists of words or pictures but everyday events. This sort of memory allows us to answer questions such as “How was the party last night?” or “What happened in the last episode of that TV show?” How can we improve it?

**Fig. 1:**
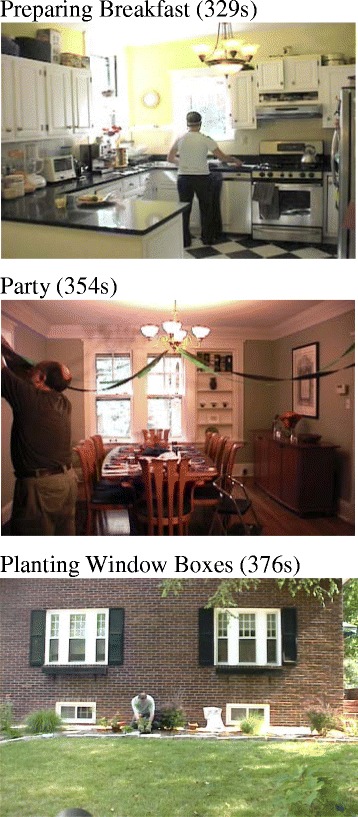
Representative stills drawn from the routine videos that show a single actor preparing breakfast with toast and eggs, arranging decorations for a party, and planting window boxes

Here, we describe a theory-driven intervention motivated by this problem. The intervention targets event encoding by inserting pauses and visual signals into a movie (see Fig. 1). These cues are placed at natural event boundaries, as determined by an empirically validated theory of event segmentation. In two experiments, this intervention improved memory for events for both younger and older adults. Interestingly, adding cues to the movies helped memory even when they were mistimed relative to the boundaries—though not as much as cues at event boundaries.

Together, these results support two implications for potential memory aids: first, using cues to guide event segmentation facilitates memory. Second, encouraging even a suboptimal segmentation is somewhat beneficial.

At the moment, this technique requires editing of videos prior to presentation. However, it should be possible to adapt it for use with televised events or instructional videos to encourage effective encoding, much like closed captioning facilitates comprehension for those with hearing deficits.

## Background

Memory problems are a frequent complaint of aging (Reid & MacLullich, 2006; Smith, Petersen, Ivnik, Malec, & Tangalos, 1996). These include “trouble remembering things that have happened recently,” “remembering where belongings are kept,” and “remembering things that have happened recently” (Jorm et al., 1997). However, there is a gap between the memory errors that are reported as concerns and the memory errors typically measured in the laboratory. Most previous studies of age-related differences in memory have depended on simple verbal or pictorial materials (Zacks, Hasher, & Li, 2000). As a result, interventions to improve memory in older adults have largely focused on techniques that are effective for remembering such simple materials. These include visual imagery, the method of loci, and semantic organization (Ball et al., 2002; Belleville et al., 2006). While such techniques may be helpful for tasks such as memorizing a shopping list or learning the names of new acquaintances, they offer limited opportunity to improve one’s ability to remember a complex naturalistic sequence of events. This sort of memory is important for performing everyday activities such as preparing meals, managing medications, and running errands.

In order to remember events well, one needs to encode them effectively. Studies of narrative comprehension and memory show that online measures of comprehension strongly predict subsequent memory for stories and movies. For example, van den Broek and colleagues developed a computational model of narrative reading that describes how concepts mentioned in a text fluctuate in activation over time, and how a concept’s history of activation is related to subsequent memory (van den Broek, Young, Tzeng, & Linderholm, 1999). They compared the model’s predictions to data from participants who were probed while reading about how much they were thinking of the concepts. The model nicely predicted the participants’ ratings. Further, the participants’ subsequent memory was related to these readings just as predicted by the model. Another example used a television comedy as the stimulus, and tracked brain activity over time as the assay of online processing (Hasson, Furman, Clark, Dudai, & Davachi, 2008). Effective online encoding was defined as brain activity that was consistent across viewers. During periods when brain activity was synchronized between two viewers, both tended to recall more.

Both comprehension and memory can be affected by aging (Madden & Dijkstra, 2009; Radvansky, 1999; Zacks, 1989). Four relevant observations emerge from the literature on the effects of age on comprehension and memory for naturalistic materials. First, whereas memory for simple arbitrary materials is almost always worse in older adults than in younger adults, memory for meaningful materials is often excellent in older adults. Second, older adults often have comprehension that is as good as that of younger adults, sometimes better. Third, comprehension in older adults benefits from being able to use previous knowledge to scaffold, constructing an online representation of a sequence of events. Finally, older adults benefit from using embodied perceptual-motor features of activity during comprehension.

These findings can be nicely accounted for by distinguishing between representations of incidental details of a set of materials and event models that represent the meaningful and salient aspects of situations described by those materials (Kintsch & van Dijk, 1978; Radvansky & Zacks, 2014). Older adults may preferentially favor event model representations at the expense of incidental details. These considerations suggest that comprehension and memory for activity by older adults could be facilitated by supporting construction of an effective representation of events as they are experienced.

One particularly important aspect of events is that they have parts that are systematically related to each other (Byrne, 2002; Cohen, 2000; Zacks & Tversky, 2001). For example, the everyday activity of making a bed includes parts such as “removing the dirty sheets” and “putting on the pillow cases.” In order to effectively remember everyday activities, it is important to effectively track this structure during encoding. In the laboratory, people’s ability to segment ongoing activity into events can be studied using a simple task in which they are asked to mark off boundaries between events while watching a movie (Newtson, 1976). Boundaries identified in this way tend to be hierarchically organized, such that boundaries of coarser-grained events line up with boundaries of finer-grained events (Zacks, Tversky, & Iyer, 2001) and tend to enclose those finer-grained boundaries (Hard, Recchia, & Tversky, 2011). Across observers, there is good agreement about the locations of event boundaries (Hanson & Hirst, 1989; Newtson, 1976). The boundaries of the largest units that individuals identify tend to align with the boundaries of smaller units, and healthy younger adults show remarkable agreement about where they believe an event boundary occurs (Kurby & Zacks, 2011). Event boundaries are associated with substantial transient changes in brain activity as measured with functional magnetic resonance imaging (MRI), whether or not individuals are actively parsing the activity with a segmentation paradigm or passively viewing the video (Zacks et al., 2001). The behavioral reliability and concomitant brain activity associated with segmentation suggest that normal perception of activity tracks event boundaries.

Previous studies have found that the ability to segment an ongoing activity into meaningful events is associated with subsequent memory for the activity (Bailey et al., 2013; Sargent et al., 2013). In these studies, event segmentation ability has been measured by comparing each individual’s segmentation with a normative sample of segmentation, a measure called segmentation agreement (Kurby & Zacks, 2011). Segmentation agreement also is associated with the ability to perform everyday actions effectively (Bailey, Kurby, Giovannetti, & Zacks, 2013). Segmentation agreement sometimes is found to be impaired in healthy older adults (Zacks, Speer, Vettel, & Jacoby, 2006; but see Sargent et al., 2013), and is impaired in early Alzheimer’s disease (Bailey, Zacks, et al., 2013; Zacks et al., 2006).

One potential mechanism for the effect of segmentation ability on subsequent memory is provided by the event horizon model (Radvansky, 2012; Radvansky & Zacks, 2014). The event horizon model is defined by five principles, of which two are relevant here. Principle 1 states that “continuous ongoing activity is segmented into discrete events, and an event model is constructed for each event.” The segmentation mechanism adopted by the event horizon model is event segmentation theory (EST; Zacks, Speer, Swallow, Braver, & Reynolds, 2007). EST proposes that comprehenders make predictions about the near future, guided by their event models. The accuracy of these predictions is monitored by the event comprehension system, and when a transient spike in prediction error occurs the event model is updated.

The second principle of the event horizon model that is relevant here is principle 5: “When several events are similar accessing any specific event model is difficult.” In naturalistic activity, effective event boundaries tend to correspond to points in time when many features of the situation are changing (Newtson, Engquist, & Bois, 1977; Zacks, Kumar, Abrams, & Mehta, 2009; Zacks, Speer, & Reynolds, 2009). Effective segmentation reduces competition during memory retrieval by binding together intervals with similar feature values, and establishing boundaries when feature values are changing. Ineffective segmentation leads to event boundaries with similar feature values on either side of the boundary, exacerbating retrieval competition.

If people who segment activity more effectively have better subsequent memory for that activity, is it possible to improve memory by intervening to improve segmentation? A very small number of previous studies hint that this may be effective. For example, one study added commercial breaks to a detective television program, either at event boundaries or at the middles of events (Boltz, 1992). When commercials were placed at event boundaries they improved memory, but when they were placed at event middles they impaired memory. In another study, participants learned how to perform assembly tasks using computer interfaces that either reinforced effective event segmentation or worked against effective segmentation (Zacks & Tversky, 2003). For example, in one experiment participants learned to assemble a tenor saxophone using an interface that either broke down the activity into events based on experts’ segmentation judgments, or simply presented the video. Compared to simply watching a video demonstration, interfaces based on effective segmentation facilitated memory and learning, whereas interfaces based on ineffective segmentation impaired performance. More recently, researchers have created customized instructional manuals based on an individual’s own event segmentation pattern to create a unique guide for the learner (Mura, Petersen, Huff, & Ghose, 2013).

### The current study

These results are tantalizing but not decisive. Moreover, none of the previous studies have tested such interventions in both younger and older populations. The results we have reviewed suggest that segmentation is an attractive target for intervention to improve adult memory, for three reasons. First, segmentation predicts subsequent memory. Second, both segmentation and memory are inefficient in older adults. Third, the limited extant data indicate that in younger adults, improving segmentation can improve memory and learning.

Therefore, in the two experiments reported here, we designed an intervention to scaffold event segmentation during encoding and tested it in younger and older adults. The basic intervention was to provide visual and auditory cues as to when the event boundaries occurred. To further support adaptive encoding, this condition also included post-viewing summaries of the videos that reviewed the event boundaries. In each experiment we compared this event boundary condition to two other conditions: an unedited condition in which the movies were provided without any cues, and an event middle condition in which cues were provided in the middle of events rather than at boundaries and the event middles were reviewed in the post-viewing summary.

We hypothesized that younger adults would show better recognition and recall memory for the unedited movies than older adults (Sargent et al., 2013). If the structural cues at event boundaries are able to modify the encoding of event structure, then older adults should show benefits in their memory in the event boundary condition. We anticipated that in the event middle condition, cues at midpoints of the video might improve memory for midpoint information in older adults, but that it would impair memory for event boundary information. To preview an unexpected result, we found that cuing event middles generally improved memory, though not as much as cuing event boundaries. One possibility is that providing even a maladaptive structure to guide encoding can be helpful in forming an integrated, durable representation (Craik & Lockhart, 1972). We will return to this issue in “General Discussion”.

## Experiment 1

Young and older adults viewed videos of everyday events with visual and auditory cues at either the event boundaries or event middles, followed by highlights of the cued segments to reinforce the structure. We examined whether structural cues at event boundaries or event middles influenced memory for the activities relative to the control condition of an unedited presentation of the video.

### Method

#### Participants

Demographic information for the sample is summarized in Table 1. We recruited 71 older adults (65% female; ages 65 − 85 years) through community advertisements and a department volunteer pool of older adults, and 61 younger adults (53% female; ages 18 − 25 years) from a university undergraduate volunteer pool. To screen for the presence of dementia, older adults were required to score less than 5 on the Short Blessed Test (Katzman et al., 1983) and less than 2 on the Ascertain Dementia 8-item questionnaire (AD-8) (Galvin et al., 2005). Additionally, older adults could not have any health conditions known to have deleterious effects on cognition, including: general anesthesia within the last year; recent head injury with loss of consciousness; a lifetime history of moderate traumatic brain injury; vascular events; untreated hypertension; neurological conditions; metabolic disorders; current or previous alcohol or substance misuse; psychiatric illness; or current use of psychotropic medications (including sleeping pills). In the older adult group, 71 individuals completed phone screening, and 41 met the criteria for and participated in the experiment. Younger adults were not screened for health conditions. Participants received $10 per hour or course credit (younger adults only) for their time and effort.

**Table 1:** Demographic information for experiment 1

	Younger adults (n = 58)		Older adults (n = 40)	
	Mean	SD	Mean	SD
Age	20.64	1.84	71.88	3.96
Years of education*	14.40	1.21	15.85	2.38
Antonym vocabulary*	.57	.21	.71	.28
Synonym vocabulary*	.64	.21	.76	.25

^*^Significant difference between older adult and younger adult groups (*p* < .0125)

#### Event movie stimuli

Participants watched movies depicting a single actor performing an everyday activity: preparing breakfast (329 s), setting up for a party (354 s), and planting window boxes (376 s). A practice movie depicting a man building a boat out of Duplos blocks (371 s) also was included. All movies were filmed in a single continuous shot at a fixed camera angle (see Sargent et al., 2013 for details). Movies were counterbalanced in three presentation conditions: (1) unedited: a continuous shot of an actor performing an everyday action; (2) event boundary: boundaries between meaningful events were marked by a bell sound, a brief slowing of the movie, and the presentation of an arrow pointing toward the object being used at the boundary; and (3) event middle: points at the temporal midpoint of meaningful events were marked using the same three cues. Examples and the full stimulus set can be viewed at https://pages.wustl.edu/dcl/stimuli-effects-cues-event-segmentation-subsequent-memory.

Event boundaries were identified based on the coarse-grained segmentation data from Sargent et al. (2013). For that experiment, participants viewed the movies and pressed a button to indicate when they judged a large meaningful event to end and another to begin. The participants’ (*n* = 208) button presses were aggregated into the probability density of identifying a coarse boundary with Gaussian kernel density estimation (3-s bandwidth) as outlined in Kurby and Zacks (2011). The peaks in the distribution of button presses for the duration of the video represent points of maximal agreement between raters about the temporal location of event boundaries.

The mean time between event boundaries was 16.74 s (SD = 5.73 s) for the 19 event boundaries extracted from preparing breakfast, 27.21 s (SD = 20.55 s) for the 12 event boundaries for setting up for a party, and 19.69 s (SD = 6.21 s) for the 17 event boundaries for planting window boxes. The first and last coarse boundary units were removed from the analysis because participants universally identify the entrance and exit of the actor in the video as event boundaries. Still pictures of the event boundaries and event midpoints were extracted for preparing breakfast (*n* = 19), setting up for a party (*n* = 12), and planting window boxes (*n* = 17) movies. The midpoints were identified as the temporal midpoint between successive event boundaries. In the one second preceding each boundary, the movie was slowed to 50% speed in order to provide a graceful transition to the freezing of the movie frame, followed by a one-second still frame at the event boundary. Concurrent with the still frame, a bell rang and the object that the actor was interacting with was cued with a red arrow. The movie resumed with another second of 50% speed and then continued at the normal rate. In some cases, the object could not be cued precisely when the event boundary was identified due to occlusion of objects (e.g., a refrigerator door blocking the view of the object to be cued). In these cases (*n* = 16 or 33% of the objects cued), the time point closest to the boundary where the target object could be seen was selected (none of the objects were cued more than 2.5 seconds from the prescribed event boundary or midpoint). The same procedure was used to cue event middles, except that editing was done at the temporal midpoint between two event boundaries.

For the event boundary and event middle conditions, summary movies (highlights) were created from the still frames drawn from either the event boundary or event middle. The frames were individually presented on the screen for 3 seconds at a time, with a 250-ms faded transition before and after the still was presented, to make the transitions between frames more natural.

To select still pictures for recognition testing, we recruited a separate sample of Amazon Mechanical Turk participants (*n* = 80) to watch the unedited movies and complete a 20-item, two-alternative forced-choice recognition task with the event boundary and event middle images following each movie. These target images were matched to lures drawn from movies depicting the same actor in the same setting but had substantial differences in the activity; for example, for the breakfast movie, items were prepared in a different order using different techniques (see Sargent et al., 2013). Lures were additionally matched to targets for visual properties such as luminance and contrast. For each movie, we selected 10 frames drawn from event boundaries (hereafter called boundary information) and 10 frames drawn from event middles (hereafter called midpoint information) for which responses were near 75% correct, so as to provide maximal sensitivity to the experimental manipulations.

#### Memory measures

Directly following presentation of each movie, memory for the movie was assessed using three measures from Zacks et al. (2006): recall, recognition and order memory. For recall, participants were instructed to type or describe in detail, the activity in the movie in the order that it occurred. Participants typed their responses on a laptop, but, if the participant preferred, the experimenter was allowed to transcribe the participant’s response for them (four older adults and one younger adult elected to do this). A maximum of 7 minutes was given to complete this task, and the time to complete the task was recorded. Participant responses were scored for accuracy by comparing them to a protocol of the precise actions that the actor in the movie performed, using the Sargent et al. (2013) adaption of the Action Coding System (Schwartz, Reed, Montgomery, Palmer, & Mayer, 1991). The protocol coded for whether each action came from a part of the movie that was never cued (uncued), cued in the event boundary condition (boundary) or cued in the event middle condition (midpoint). Recall memory was scored as the proportion of correct uncued, boundary, and midpoint information (inter-rater Kappa score = .89 (*p* < .0001), 95% CI .80, .98).

Following recall, participants completed a test of recognition memory consisting of 20 trials with 10 boundary and 10 midpoint target still pictures taken from the movies randomly matched to lure pictures from a foil movie. For each trial, participants viewed both a target and a lure and were instructed to select the picture that came from the movie. Recognition memory was scored as the proportion of trials answered correctly for each movie. For order memory, the experimenter laid in front of the participant 12 laminated pictures from the movie, printed on 10 cm × 15 cm index cards, in a randomized order. Participants were instructed to arrange the cards in the correct chronological order. Order memory performance was scored as an error measure, which was the mean absolute deviation from the correct position for each picture; in this case, lower scores on the task meant better performance. Time to complete the task was also recorded.

#### Vocabulary test

A vocabulary test was administered to compare younger and older adults on their general verbal abilities. Vocabulary was measured by having participants complete a computerized synonym and antonym vocabulary test (Salthouse, 1993). For this task, a vocabulary word was presented along with four other word choice options. For synonyms, participants were instructed to select the word that was closest in meaning to the vocabulary word. For antonyms, participants selected the word opposite in meaning. Both the antonym and synonym tasks consisted of ten trials.

#### Procedure

All participants provided informed consent consistent with the Institutional Review Board (IRB) standards at Washington University in St. Louis. After participants provided their consent, we assessed visual acuity by having participants read lines of letters from a Snellen eye chart to the experimenter to verify participants had sufficient visual acuity to view the movies. Participants were then accompanied into a quiet testing chamber with the experimenter. They were comfortably seated approximately 66 cm from the computer screen (2009 iMac with a 20 inch-wide screen, and 1690 × 1050 resolution). All movies were presented at a 720 × 480 aspect ratio. The movies, instructions, and recognition tasks were all presented using E-Prime software (Schneider, Eschman, & Zuccolotto, 2002).

To practice the primary experimental tasks, participants watched the practice movie. The movie was modified to present the cuing in the same manner as either the edited event boundary or event middle presentations. Participants were always given the standard prompt before viewing movies edited at the event boundary or event middle: “The video has been modified to draw attention to important parts of the video with an arrow, bell, and slowed-down at certain times.” Participants were always told to “pay close attention because afterwards, your memory for the video will be tested.” Following presentation of the practice movie, participants viewed the movie displaying the summary of the cued frames. Participants were given the standard prompt to: “pay close attention to the summary videos as an opportunity to review important parts of the movie that you watched.” Participants then completed a practice recall trial for the movie.

After completing the practice recall task, participants were presented with an example of an “ideal” recall response, drawing attention to the level of detail and type of information that could be noted such as descriptions of the color of items or the way that that actor interacted with the objects. Participants then completed a practice recognition test.

After the practice session, participants proceeded to the experimental movies. For the unedited condition, participants were told that they would be watching a movie without a summary presented afterwards. For the event boundary and event middle conditions, participants were given the standard prompts for the editing and the summary movie that followed. Participants always completed the recall task, followed by the recognition, and the order memory.

Following these experimental tasks, participants completed a familiarity questionnaire about the frequency that they prepare breakfast, set up for a party, and plant flowers in a garden. Participants were debriefed and asked to provide feedback about the experiment. Participants were asked: (1) how helpful he or she found the modifications to the movie, and the utility of the summary movies, (2) which condition he or she found the most impactful, and (3) whether he or she employed any particular strategies when trying to remember the movies.

#### Data processing and exclusion

Outliers in recognition response time were identified as those responses faster than 500 ms or more than 3 SD slower than each participant’s mean response time; these were discarded (1.54% of observations for older adults, 1.22% for younger adults). Four participants with mean recognition accuracy below .60 were identified as outliers on accuracy and excluded (one older, three younger participants), yielding a final sample of 40 older and 58 younger adults.

The experiment was adequately powered to detect small main effects, and small to medium interactions. The primary dependent variables met assumptions for analysis of variance (ANOVA), and were approximately normally distributed (|skewness| <2.0, |kurtosis| <2.0). We used R (R Core Team, 2013) and the lme4 package (Bates, Mächler, Bolker, & Walker, 2015) to estimate linear mixed effects analyses with reduced maximum likelihood. Linear mixed models included random effects of subjects and movies, and fixed effects of age group (younger adult, older adult), presentation condition (unedited, event middle, event boundary), and information type (midpoint, boundary). To test the statistical significance of the main effects and to perform post hoc tests of pairwise differences, we used the lmerTest package (Kuznetsova, Brockhoff, & Christensen, 2015), using Satterthwaite's approximation for degrees of freedom.

### Results and discussion

#### Recall memory

**Fig. 2:**
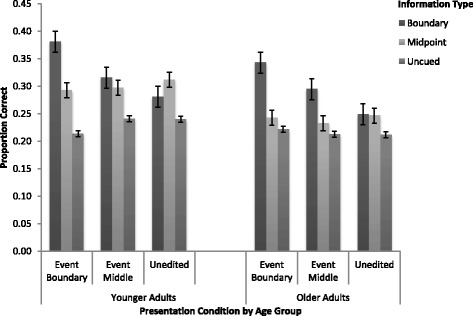
Experiment 1: recall accuracy (+/-SE) by condition and type of information

As is shown in Fig. 2, younger adults (mean (M) = 0.29, SD = 0.14) recalled more than older adults (M = 0.25, SD = 0.14), resulting in a significant fixed effect of group, *F* (1, 96) = 5.16, *p* = .025, *d* = 0.25. There was also a fixed effect of presentation condition, *F* (2, 765) = 3.47, *p* = .031, indicating that recall accuracy was better in the event boundary condition (M = 0.29, SD = 0.16) compared to the unedited presentation (M = 0.26, SD = 0.13), *t* (765) = -2.58, *p* = .01, *d* = 0.17, and marginally better than the event middle condition (M = 0.27, SD = 0.14), *t* (765) = -1.71, *p* = .09, *d* = 0.11. The event middle and unedited conditions did not differ, *t* (765) = -0.87, *p* = .38, *d* = 0.07. There was a significant fixed effect of information type, *F* (2, 768) = 39.75, *p* < .001. Recall accuracy was better for boundary information (M = 0.31, SD = 0.18) than uncued information (M = 0.23, SD = 0.08), *t* (766) = 8.95, *p* < .001, *d* = 0.65, and midpoint information (M = 0.28, SD = 0.14), *t* (768) = 4.12, *p* < .001, *d* = 0.23. Recall accuracy for midpoint information was better than for uncued information, *t* (768) = 4.83, *p* < .001, *d* = 0.45.

We did not find significant interactions between group and presentation condition, *F* (2, 765) = 0.24, *p* = .78, or group and information type, *F* (2, 768) = 2.67, *p* = .07. There was a significant interaction between presentation condition and information type, *F* (4, 765) = 6.86, *p* < .001. We followed up this effect separately for each information type, and only boundary information was recalled differently between presentation conditions, *F* (2, 190) = 14.03, *p* < .001. Consistent with our hypotheses, boundary recall accuracy was better in the event boundary condition (M = 0.37, SD = 0.18) relative to the unedited condition, (M = 0.27, SE = 0.16), *t* (190) = -5.26, *p* < .001, *d* = 0.58, and compared to the event middle condition (M = 0.31, SE = 0.18), *t* (190) = -3.19, *p* = .002, *d* = 0.33. Boundary recall was also better in the event middle condition compared to the unedited condition, *t* (190) = -2.06, *p* = .04, *d* = 0.23.

#### Recognition memory

**Fig. 3:**
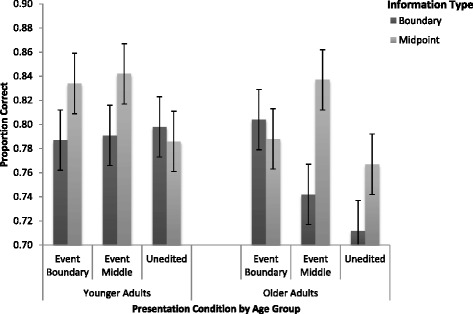
Experiment 1: recognition accuracy (+/-SE) by group and presentation condition

As shown in Fig. 3, younger adults (M = 0.81, SD = 0.15) had better recognition performance than older adults (M = 0.78, SD = 0.16), resulting in a significant fixed effect of group, *F* (1, 96) = 4.05, *p* = .047, *d* = 0.20. The fixed effect of presentation condition was significant, *F* (2, 478) = 4.93, *p* = .008. Cuing at the event boundary (M = 0.80, SD = 0.15) improved recognition memory compared to the unedited presentation (M = 0.77, SD = 0.16), *t* (478) = -2.68, *p* = .008, *d* = 0.22, as did cuing at the event middle (M = 0.81, SD = 0.16) compared to the unedited presentation, *t* (478) = -2.76, *p* = .006, *d* = 0.22. Contrary to predictions, the event boundary and event middle conditions did not differ significantly, *t* (478) = 0.07, *p* = .94, *d* = 0.01.

Midpoint information (M = 0.81, SD = 0.15) was recognized better than boundary information (M = 0.78, SD = 0.16), resulting in a significant fixed effect of information type, *F* (1, 478) = 11.06, *p* < .001, *d* = 0.23. There were no significant interactions between group and presentation condition, *F* (2, 478) = 0.94, *p* = .39, or group and information type, *F* (1, 478) = 0.52, *p* = .477. The interaction between presentation condition and information type approached significance, *F* (2, 478) = 2.71, *p* = .067.

There was a three-way interaction between group, presentation condition, and information type, *F* (2, 478) = 3.25, *p* = .039 that we examined separately by group. For younger adults, the interaction between presentation condition and information type was not significant, *F* (2, 283) = 2.18, *p* = .11. However, for older adults the interaction between presentation condition and information type was significant, *F* (2, 193) = 3.49, *p* = .032. For older adults, cuing the event boundaries enhanced recognition of boundary information (M = 0.80, SD = 0.16) relative to boundary information in the unedited condition (M = 0.71, SD = 0.18), *t* (76) = 2.68, *p* = .009, *d* = 0.53, and marginally better than boundary recognition when cuing at event middles (M = 0.74, SD = 0.16), *t*(76) = 1.92, *p* = .059, *d* = 0.38, whereas boundary information was not improved by event middle cuing alone relative to the unedited condition, *t* (76) = 0.77, *p* = .45, *d* = 0.17. By contrast, cuing at event middle (M = 0.84, SD = 0.15) improved older adults’ recognition of midpoint information compared to the unedited condition (M = 0.77, SD = 0.16), *t* (76) = 2.51, *p* = .01, *d* = 0.46, and marginally compared to the event boundary condition (M = 0.79, SD = 0.15), *t* (76) = -1.78, *p* = .08, *d* = 0.32. The random effects of subjects, χ^2^ (1) = 22.0, *p* < .001, and movie type, χ^2^ (1) = 98.9, *p* < .001, were significant.

The order memory task was not included in the analyses because performance on this measure was uniformly excellent, with minimal variability. For both older and younger adults, the modal order error rate was 0 (older adults: M = 0.76, SD = 0.93; younger adults: M = 0.23, SD = 0.47).

#### Discussion

We hypothesized that cuing at event boundaries would improve memory, and that this might reduce age differences in memory. In both recognition and recall, cuing at event boundaries improved memory over the control condition, as predicted. For recall memory, cuing at the event boundary enhanced boundary accuracy more so than the other conditions. This supports the idea that intervening with event encoding can selectively affect how an activity is encoded for memory. It also is consistent with the proposal from EST that event boundaries anchor one’s organization of events in memory. However, we found that cuing at event middles also improved recognition memory somewhat, and for neither recognition nor recall was there a striking advantage for the boundary cuing condition over the middle cuing condition. One possibility is that because both cuing conditions involved visual cuing of the target object, recognition represented lower-level familiarity with target objects as opposed to facilitating a richer memory representation for the activity. Another possibility is that pausing the video and giving participants the time to reflect was beneficial independent of when the pauses were taken. Finally, it could have been that the cuing conditions were most efficacious because they both contained summary videos that provided multiple exposures to the target objects. We addressed these possibilities in experiment 2.

We found that boundary information was better recalled than midpoint and uncued information, consistent with other investigations (Baldwin & Baird, 2001; Schwan & Garsoffky, 2004; Swallow, Zacks, & Abrams, 2009). However, to our surprise, pictures from event midpoints were better recognized than those from boundaries. At first glance this is surprising because previous studies have found that pictures from event boundaries are more distinctive and better recognized (e.g., Newtson & Engquist, 1976). However, we selected pictures for the recognition test based on pilot testing, choosing boundary and midpoint items with intermediate baseline recognition performance in order to optimize the ability to detect effects of the cuing manipulation. A likely possibility is that our pilot testing overcame any general advantage for recognition of boundary items, and perhaps through unlucky selection converged on items that ran strictly counter to it.

As expected, younger adults had better recognition accuracy and recall memory than older adults. Both groups benefited from the cuing manipulations; that is, the intervention did not rescue the older adults’ poorer performance.

## Experiment 2

In experiment 2, we first sought to replicate the benefits of cuing at event boundaries for subsequent memory in younger and older adults. Second, we modified the cuing procedure to reduce the incidental advantages for the cuing conditions over the control condition, by eliminating the arrows cuing task-relevant objects and by adding to the control condition an opportunity for participants to review the activity after encoding. By doing so, we aimed to better answer whether cuing event structure *per se* benefited memory encoding.

### Method

#### Participants

Demographic information is summarized in Table 2. The recruitment procedures were identical to those in experiment 1. In the older adult group (ages 65–85 years), 74 individuals completed phone screening, and 43 met the criteria for and participated in the experiment; 41 younger adults participated in the experiment.

**Table 2:** Demographic information for experiment 2

	Younger adults (*n* = 40)		Older adults (*n* = 40)	
	Mean	SD	Mean	SD
Age	19.85	1.88	74.23	5.59
Years of education*	13.78	1.56	15.81	1.95
Antonym vocabulary*	0.62	0.22	0.73	0.25
Synonym vocabulary*	0.62	0.20	0.75	0.25

^*^Significant difference between older adult and younger adult groups (*p* < .0125)

Younger adults (70% female) had fewer years of education, and performed more poorly on measures of vocabulary than older adults (66% female). A comparison of Tables 1 and 2 indicates that participants from Experiment 1 and 2 were similar in age, education, and vocabulary.

#### Design, materials and procedure

The design, materials, and procedure were identical to those in experiment 1 except for two changes to the stimulus materials, which were designed to make the three conditions as similar as possible except for the event structure manipulation. First, in the movies in experiment 1, the event boundary and event middle conditions included arrows cuing task-relevant objects. Calling attention to relevant objects could improve event encoding via mechanisms other than segmentation, and therefore, these were removed. Second, in experiment 1 the event boundary and event middle conditions included post-viewing summaries, but the control condition did not. For experiment 2, post-viewing summaries were created for the control condition by making sped-up versions of the movies. Presentation speed of each control summary movie was set to match the duration of the corresponding highlight movies for the event boundary and event middle conditions (e.g., breakfast summary = 68 s, full movie = 328 s, summary movie presented at an increased speed of 450%). Participants were oriented to the different presentation styles using the same practice movie from experiment 1, followed by the highlight movie and the summary movie presented at a faster speed.

As in experiment 1, participants viewed a movie edited at the event boundary, the event middle, and unedited, in counterbalanced order. Prior to viewing each movie in the experiment, participants were always informed of the type of movie that they would be viewing (edited or unedited) and the type of post-viewing summary movie that they would be viewing (highlights or sped-up presentation). Movies edited at the event boundary or event middle were always followed by the corresponding highlight movies, and the unedited movies were always followed by the corresponding sped-up presentation. The same procedure from experiment 1 was followed for investigating memory for the movies. As before, participants were offered the option of typing their recall protocols themselves or dictating to the experimenter. Two older adults opted to have the experimenter type for them; all younger adults typed for themselves.

#### Data processing and exclusion

Recognition trials were trimmed of outliers in the same way as in experiment 1, resulting in the exclusion of 0.91% of trials in the older adults and 1.54% of trials in the younger adults. One older participant’s data were not recorded due to experimenter error. Two older adults and one younger adult with mean recognition accuracy below .60 were excluded from analyses, yielding a final sample of 40 older and 40 younger participants.

The experiment was adequately powered to detect small main effects, and small to medium interactions. The primary dependent variables met assumptions for ANOVA, and were approximately normally distributed (|skewness| < 2.0, |kurtosis| < 2.0).

### Results

#### Recall memory

**Fig. 4:**
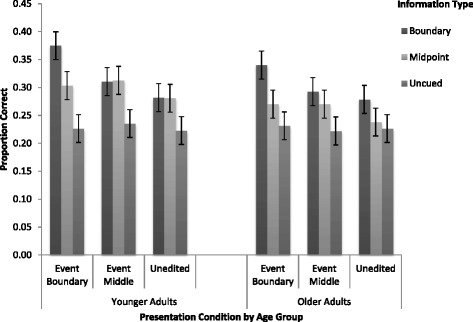
Experiment 2: recall accuracy (+/-SE) by condition and type of information

Unlike experiment 1, younger adults (M = 0.28, SD = 0.13) did not recall significantly more than older adults (M = 0.26, SD = 0.13), *F* (1, 78) = 1.94, *p* = .17, *d* = 0.15 (see Fig. 4). As in experiment 1, there was a fixed effect of presentation condition, *F* (2, 622) = 6.01, *p* = .003, such that recall accuracy was better in the event boundary condition (M = 0.29, SD = 0.14) compared to the unedited presentation (M = 0.25, SD = 0.11), *t* (622) = 3.47, *p* < .001, *d* = 0.29. Event boundary recall accuracy was marginally better than the event middle condition (M = 0.27, SD = 0.14), *t* (621) = 1.68, *p* = .09, *d* = 0.12, and the event middle condition was marginally better than the unedited condition, *t* (622) = 1.79, *p* = .07, *d* = 0.15. As in experiment 1, there was a significant fixed effect of information type, *F* (2, 622) = 37.99, *p* < .001. Recall accuracy was better for boundary information (M = 0.31, SD = 0.16) than uncued information (M = 0.23, SD = 0.07), *t* (621) = 8.45, *p* < .001, *d* = 0.68, and midpoint information (M = 0.28, SD = 0.12), *t* (621) = 3.42, *p* < .001, *d* = 0.23. Recall accuracy for midpoint information was also better than uncued information, *t* (621) = 5.24, *p* < .001, *d* = 0.52.

As in experiment 1, we did not find significant interactions between group and presentation condition, *F* (2, 622) = 0.09, *p* = .92, or group and information type, *F* (2, 622) = 1.84, *p* = .16. (Fig. 4). There was a significant interaction between presentation condition and information type, *F* (4, 622) = 3.10, *p* = .015, that we investigated separately for each information type, as in experiment 1. The interaction was driven by the differential recall of boundary information between presentation conditions, *F* (2, 154) = 8.69, *p* < .001, relative to the recall of midpoint, *F* (2, 154) = 2.97, *p* = .054, and uncued information, *F* (2, 154) = 0.16, *p* = .86. Consistent with the hypotheses, boundary recall accuracy was better in the event boundary condition (M = 0.36, SD = 0.17) relative to the unedited condition, (M = 0.28, SE = 0.14), *t* (154) = 4.01, *p* < .001, *d* = 0.49, and compared to the event middle condition (M = 0.30, SE = 0.17), *t* (154) = 2.99, *p* = .003, *d* = 0.33. Boundary recall did not differ between the event middle condition compared to the unedited condition, *t* (154) = 1.02, *p* = .31, *d* = 0.13. The random effects of subjects, χ^2^ (1) = 59.6, *p* < .001, and movie, χ^2^ (1) = 37.8, *p* < .001 were significant.

#### Recognition memory

**Fig. 5:**
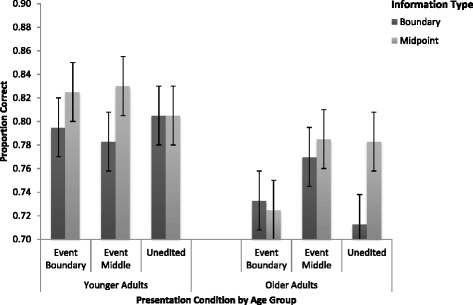
Experiment 2: recognition accuracy (+/-SE) by presentation condition and group

As shown in Fig. 5, younger adults (M = 0.81, SD = 0.14) had better recognition accuracy than older adults, (M = 0.75, SD = 0.17), resulting in a significant fixed effect of group, *F* (1, 78) = 10.32, *p* = .002, *d* = 0.36. Contrary to hypotheses and to the findings of Experiment 1, the fixed effect of presentation condition was not significant, *F* (2, 388) = 1.11, *p* = 0.33, indicating that the cuing manipulation did not have a reliable overall influence on recognition memory. Midpoint pictures (M = 0.79, SD = 0.15) were once again recognized significantly better than boundary pictures (M = 0.77, SD = 0.16), *F* (1, 388) = 4.56, *p* = .03, *d* = 0.17. There were no significant interactions between group and presentation condition, *F* (2, 388) = 1.49, *p* = 0.22, or group and information type, *F* (1, 388) = 0.00, *p* = .99, or the three-way interaction, *F* (2, 388) = 2.11, *p* = .12. The random effects of subjects, χ^2^ (1) = 20.0, *p* < .001, and movie, χ^2^ (1) = 67.0, *p* < .001 were significant.

As in experiment 1, performance in the order memory task was excellent and not sufficiently variable to permit further analysis. For both older and younger adults, the modal order error rate was 0 (older adults: M = 0.87, SD = 0.94; younger adults: M = 0.25, SD = 0.51).

#### Discussion

The recall results of experiment 2 replicated and clarified the patterns observed in experiment 1. The cuing manipulation affected recall memory performance, and the pattern was similar to experiment 1: recall was significantly better for the boundary cuing condition than the control condition, and the middle cuing condition was in between. This suggests that not all of the benefits of the boundary cuing condition are due to the visual cuing of task-relevant objects, nor to the post-viewing review, because in this experiment no visual cues were present and a post-viewing review was added to the control condition. We did not observe significant effects of cuing on recognition memory, unlike in experiment 1. We do not see a ready explanation for this difference; it could simply be a false negative, or possibly reflects that the recognition test is a less sensitive measure than the recall test.

Younger adults again remembered more than older adults, but this was significant only for recognition memory. As in experiment 2, we found that midpoint pictures were better recognized than boundary pictures, suggesting that whatever factors led to this surprising pattern are stable for these materials. Also, as in experiment 1, boundary information was better recalled than midpoint information. Finally, experiment 2 replicated the finding of experiment 1 that cuing at event boundaries selectively enhanced recall of information from those event boundaries.

In summary, the results of experiment 2 replicated those of experiment 1 with some minor differences. They thus help to rule out incidental explanations for the benefits of cuing event boundaries, and support the proposal that scaffolding event structure during encoding improves memory. The fact that recall memory after the event middle condition was again intermediate between the event boundary condition and the control condition, and was not significantly different from either, is a little vexing. This pattern is most consistent with the possibility that the event cuing manipulation was effective for two reasons: first, for the reason just stated, that scaffolding event structure facilitates memory; and second, because pauses during encoding are beneficial wherever they occur. From the point of view of practical implications, either mechanism supports the use of the event boundary cuing procedure to improve memory. From the point of view of psychological mechanisms, more work is needed to tease apart these two potential contributions.

### General discussion

In both of these experiments, we found that cuing event boundaries facilitated subsequent memory compared to a standard intentional encoding control. The benefits accrued particularly to the cued boundaries but also generalized to information from other time points. This finding supports views of event memory that propose that the segmentation of ongoing activity into events determine the structure of subsequent episodic memory (Ezzyat & Davachi, 2011; Radvansky & Zacks, 2014). It also has potential practical significance for the design of information interfaces.

Of further potential practical significance, we also found that the cuing procedure was somewhat effective even when cues were placed in the “wrong” locations, in the middle of events. The event middle condition, in which event boundaries were cued at locations determined to fall in the middles of cognitive natural events, led to memory performance that was intermediate between the event boundary condition and the unedited control condition and for the most part was not statistically significantly different from either. This suggests that giving an opportunity to pause and take stock of an ongoing activity benefits event encoding even when the pause is not time-locked to the event structure. This could give an opportunity to process event structure as such, but just as easily could benefit memory by allowing one to process other aspects of the activity such as objects or goals.

These findings are consistent with a recent study that examined memory for word lists and narrative texts in and around the introduction of shifts in the event structure (Pettijohn, Thompson, Tamplin, Krawietz, & Radvansky, 2016). In that study, event structure was manipulated by asking participants to walk through a doorway midway through learning a word list, or view the second half of a word list on a different computer screen window. Relative to the control condition, structuring the event encoding improved memory for the word list overall, though it did not selectively improve memory for the words around the event boundary.

#### Potential mechanisms

As described in “Introduction”, the event horizon model proposes a mechanism for these effects (Radvansky & Zacks, 2014). In the present experiments, the cuing conditions introduced salient feature changes at event boundaries or event middles: visual slowing, a tone, and—in experiment 1—an arrow cuing an object. This is proposed to induce a transient spike in prediction error, resulting in segmentation at the point of the cue (Zacks et al., 2007). In the boundary condition, induction of a cue at a natural event boundary should facilitate effective encoding by inducing long-term memory representations that optimally reflect the underlying structure of the activity. Such representations reduce retrieval competition by grouping together intervals with similar relevant feature values, and separating intervals with differences in relevant features. In the event middle condition, inducing prediction errors in the middle of what would naturally be an event conflicts with effective segmentation, resulting in memory representations that compete more in long-term memory.

Other mechanisms could potentially contribute to these effects. Cuing event structure may also have facilitated the use of top-down knowledge and the inference of goals and causation. For example, prior knowledge about the kinds of goals and intentions enacted in a kitchen leads to expectations about meaningful units of activity. In the video of an actor preparing breakfast, our participants likely had a range of expectations about what would occur when an egg was cracked and placed into a frying pan. Expert dancers use their prior knowledge to parse ongoing dance movements differently to novice observers (Bläsing, 2015). Viewers also incorporate features such as goal-directed changes in behavior such as the shift in intentions when footballers switch from offensive schemes to defensive postures (Huff, Papenmeier, & Zacks, 2012). Therefore, reinforcing the event structure may help to consolidate the inherent causality and goal structure of ongoing activity and allow individuals to efficiently parse ongoing activity.

The strategy used here has clear limitations that should be explored in further research. First, from a practical perspective it would be helpful to test additional control conditions to investigate which features of cuing led to benefits in the event middle condition. One possibility we have already noted is that simply encouraging viewers to pause and consolidate their ongoing comprehension is adaptive for memory encoding. To explore this possibility, it would be of interest to test a condition in which cues are placed randomly, or in which viewers are encouraged to pause the video at times of their choosing. Another possibility is that the post-viewing slide show is a particularly effective way to rehearse a sequence of events. In experiment 1, the unedited condition had no post-viewing review; in experiment 2, we added a fast-motion video review to that condition. This had the advantage of not selecting some moments in time for rehearsal, but fast-motion video differs from slide shows on several dimensions. A slide show of randomly selected still pictures would be a welcome addition for future research.

Second, there are stable individual differences in segmentation (Speer, Swallow, & Zacks, 2003), which could reflect differences in what would best function as effective segmentation for each individual. Given the differing knowledge and interests of individuals, it is possible that there is no single segmentation that will be best for all viewers. And finally, if a viewer is experiencing difficulty segmenting due to disruptions in perceptual processing, error monitoring, or event model maintenance, then the intervention may not be able to fully rescue their performance. Such disruptions may be present in age-related neurodegenerative diseases including Alzheimer’s disease (Bailey, Zacks, et al., 2013; Zacks et al., 2006) and Parkinson’s disease (Fernandino et al., 2013; Zalla et al., 1998; but see Schiffer et al., 2015).

If individual or group differences in segmentation ability are meaningful, future studies may seek to capitalize on customized event boundaries as has been done in the Instructions Based on Event Segmentation tool (Mura et al., 2013). This tool allows individuals to create instruction manuals for complex activities based on normative boundary points in the task. Individuals can create their own instructional materials based on how they parsed an activity at initial viewing with still images and instructions. This type of approach may be able to minimize the variability in event perception and comprehension to maximize memory for the activity. It may have also been the case that simply pausing, in and of itself, encourages integration that is effective. As discussed earlier, working memory is related to event segmentation, and perhaps simply pausing the video at regular intervals allows for better segmentation through indirect mechanisms related to processing speed.

#### Addressing complaints of aging

Encouragingly, our modifications to the presentation of everyday videos were effective for both younger and older adults, and this suggests that both groups have the necessary cognitive resources at encoding to benefit from the scaffolding manipulation. In this regard, our findings align with an investigation of event processing in a recent study with similar age groups (Radvansky, Pettijohn, & Kim, 2015). As younger and older adults passed through a virtual doorway with an object in their hand, they were equally likely to forget the name of the object. An event-specific explanation is that working memory is updated at this boundary and the prior event model with the object is discarded in favor of the updated model that is ready to encode new information. In many instances aside from event segmentation, age differences in event processing can be minimal (e.g., Magliano, Kopp, McNerney, Radvansky, & Zacks, 2012). Rather, processes known to decline with age, such as source monitoring and managing interference, may contribute to age-related differences when older adults have to retrieve multiple event models from long-term memory (Radvansky, 2005). Given that working memory is reliably associated with event segmentation ability, targeting these executive function skills may improve event memory.

In the present sample the younger and older adults were both highly educated, and it may have been the case that older adults with less cognitive reserve would have benefited more from our intervention. Older adults were carefully screened for health-related conditions that could impact cognition, such as diabetes or poorly controlled hypertension (Waldstein & Elias, 2015). These vascular risk factors impact the majority of older adults in North America, and so our sample may not have been representative of the population, which could have mediated age-related differences in cognition.

Another issue to consider is that we may have observed larger effects of our experimental condition if we had investigated older adults with cognitive impairments who have true differences in their identification of normative event boundaries and subsequent memory (Bailey, Kurby, et al. 2013). The older adults in our study did not have cognitive complaints that lead individuals to pursue medical investigation, and so we speculate that those who are truly in need of structural support at encoding may benefit even more.

Future research may also extend the cuing paradigm to videos that are less familiar and require even more scaffolding of the inherent structure, such as novel naturalistic actions (Gold & Park, 2009). A final question surrounds the nature of the memory representation over a delay, a question we are currently testing (Flores, Bailey, Eisenberg, & Zacks, in press). Previous findings indicate that boundary information is psychologically privileged, and better remembered than other elements of an event. Thus, it is possible that the advantages of cuing event boundaries grow with delay.

#### Conclusion

People who suffer memory impairments due to age, disease, or injury tend to present to clinics with concerns such as trouble in learning how to use new tools or gadgets, and concerns about forgetting appointments. These are concerns about memory, to be sure, but more fundamentally they are concerns about memory for events. Many aspects of event memory are shared with memory for other sorts of materials, but some aspects of event memory reflect the unique structure of a sequence that unfolds within a spatiotemporal framework (Radvansky & Zacks, 2014). The present findings suggest that scaffolding the encoding of event structure may facilitate event memory. This speaks to the unique features of event memory, and may be of practical use in providing a means to improve memory of the sort that people find most salient in their daily lives.

## Abbreviations

ANOVA: analysis of variance
EST: event segmentation theory
